# Local Anesthetic Plasma Concentrations as a Valuable Tool to Confirm the Diagnosis of Local Anesthetic Systemic Toxicity? A Report of 10 Years of Experience

**DOI:** 10.3390/pharmaceutics14040708

**Published:** 2022-03-26

**Authors:** Camille Riff, Axel Le Caloch, Julien Dupouey, Laurent Allanioux, Marc Leone, Olivier Blin, Aurélie Bourgoin, Romain Guilhaumou

**Affiliations:** 1 Laboratoire de Pharmacologie Clinique, Service de Pharmacologie Clinique, APHM, INSERM, Institut Neurosciences Système, UMR 1106, Aix Marseille Université, 13005 Marseille, France; camille.riff@gmail.com (C.R.); axel.le-caloch@ap-hm.fr (A.L.C.); julien.dupouey@labosud-provence.fr (J.D.); laurent.allanioux@ap-hm.fr (L.A.); olivier.blin@ap-hm.fr (O.B.); 2 Service d’Anesthésie-Réanimation, Hôpital Nord, Assistance Publique des Hôpitaux de Marseille, APHM, Aix Marseille Université, 13005 Marseille, France; marc.leone@ap-hm.fr (M.L.); aurelie.bourgoin@ap-hm.fr (A.B.)

**Keywords:** local anesthetics systemic toxicity, concentration, pharmacokinetics, overdose, intravascular injection

## Abstract

Background: Local anesthetic systemic toxicity (LAST) has been reported as a serious complication of local anesthetic (LA) peripheral injection. The signs and symptoms of LAST are highly variable, and the challenge remains to confirm its diagnosis. In this context, the determination of LA plasma concentration appears as a valuable tool to confirm LAST diagnosis. The aims of this study were to describe observed LA concentrations in patients suspected with LAST and their contribution to diagnostic confirmation. Methods: We retrospectively reported suspected LAST in patients for which at least one plasma LA concentration was determined to confirm diagnosis of LAST. Data collection came from our pharmacological laboratory’s database. Clinical signs and symptoms of toxicity, their onset time and observed LA concentrations were used to confirm LAST diagnosis. Results: 33 patients who presented with suspected LAST after ropivacaine and/or lidocaine administration were included. Prodromal symptoms were observed in 13 patients. Isolated central nervous system (CNS) toxicity occurred in 11 patients, and combined CNS and cardiovascular toxicity occurred in 12. One, two or three venous plasma samples were performed in 11, 3 and 19 patients, respectively. Toxic plasma LA concentrations were observed in three patients, receiving peripheral LA injection using lidocaine (16.1 µg/mL) and ropivacaine (4.2 and 4.8 µg/mL). Conclusion: This study presents an important biological and clinical dataset of patients who presented with suspected LAST. Plasma LA concentrations could bring valuable information in the diagnosis of LAST but requires rigorous sample protocols.

## 1. Introduction

Local anesthetics (LA) have been used for years to perform regional anesthesia due to the extended duration of analgesia and its good tolerability. Nevertheless, local anesthetic systemic toxicity (LAST) can occur over the injection of ester or amino-amides local anesthetics after their passage through the blood [1,2,3,4]. Clinical presentation of LAST consists of prodromal symptoms such as metallic taste, tinnitus, disorientation, logorrhea and dizziness, followed by seizures. At higher plasma drug concentrations, neurological depression and cardiac toxicity can occur [3,5,6]. However, the symptomatology is highly variable and atypical presentations are common [6]. In this context, diagnosing LAST remains a significant problem in medical practice; therefore, improving its understanding and management is a current topic that needs to be discussed [1]. Inadvertent intravascular administration and excessive absorption due to overdose are the major mechanisms involved in LAST apparition [3,4,7,8]. Consequently, it is mandatory for clinicians to respect the prevention guidelines when administering LA, i.e., adapting proper aspirating techniques and complying with the maximum recommended dosage. Delay and clinical signs are usually crucial in diagnosing LAST and in discriminating between the two mechanisms. Plasma concentration-time profiles of LA are markedly affected by factors such as injection site, LA concentrations and dose. Therefore, the time for LA to reach systemic circulation depends on the injection site and affects the delay of clinical events [3]. However, due to highly variable clinical presentations and variable delay to observe LA toxicity, confirming the diagnosis of LAST based on clinical observations could be difficult. In this context, the determination of LA plasma concentrations has been proposed as a valuable tool to confirm LAST diagnosis and etiology [8,9,10]. The aims of this retrospective observational study are then to describe: (1) observed LA concentrations in cases of suspected LAST and (2) their contribution to diagnostic confirmation.

## 2. Materials and Methods

A multicentric, retrospective, observational study was carried out in Marseille University Hospitals (APHM, Marseille France) on hospitalized patients from 2011 to 2021. The study was approved by the Institutional Research Ethics Board (Aix-Marseille University) as a retrospective, non-interventional and anonymous study (number 2018-14-03-008).

Inclusion criteria were neurologic or cardiac toxicity occurring after local or regional anesthesia performed by ropivacaine or lidocaine for pain management or surgical procedures, and at least one venous blood sample drawn following LA administration to determine LA concentration. Exclusion criteria were children under 16 years of age, local or regional anesthesia performed by other LA (plasma concentration not determined in our hospital laboratory), administration of LA via continuous infusion and incomplete description of the type of block or clinical symptoms. The following data were collected from patient medical records: patient’s age; weight and comorbidities; clinical setting; surgical procedure; type of anesthetic procedure; administered LA; dosage and concentration; LAST onset time; and signs and symptoms of toxicity. Treatment of LAST, outcome of LAST, LA blood concentrations and sampling time (recorded by nursing staff) were registered as well.

Local anesthetic plasma concentrations were measured using two distinct analytical methods validated according to EMA guidelines [11], during the studied period: 1. Gas chromatography coupled to nitrogen–phosphorus detector method before December 2018 [12]; 2. Liquid chromatography coupled to tandem mass spectrometry since December 2018. The limit of quantification of the two methods was 0.05 ng/mL, with satisfactory accuracy and precision (bias and coefficient of variation <20%, respectively). The cross-validation of the applied analytical methods has been verified with a bias <15% between the two methods (*n* = 20 samples analyzed by both analytical methods).

LA concentrations were compared to toxic thresholds determined in healthy subjects (6–7 μg/mL and 3–3.5 μg/mL for lidocaine and ropivacaine, respectively) [5,13,14]. A brief delay to observe clinical events was in favor of accidental intravascular injection. Excessive vascular absorption and potential overdose were suggested in the case of delayed symptoms and if LA plasma concentration went above the toxic threshold. For all patients, medical history and extended evaluations were considered by practitioners in order to exclude other diagnosis (anaphylaxis, side effects from medications, which were co-administered to facilitate procedural sedation, syncopal events, etc.).

Since October 2013, a protocol of three plasma samples drawn at defined times was proposed by our pharmacological laboratory in case of suspected LAST. An initial plasma sample was drawn shortly after toxic events, followed by two others: one and three hours later. A rapid decrease in LA concentrations was in agreement with inadvertent intravascular injection. Conversely, in the case of peripheral block injection, a slow decrease in LA concentrations is expected, with a plateau in the first few hours post-injection [15,16,17,18,19,20,21,22].

## 3. Results

**Figure 1:**
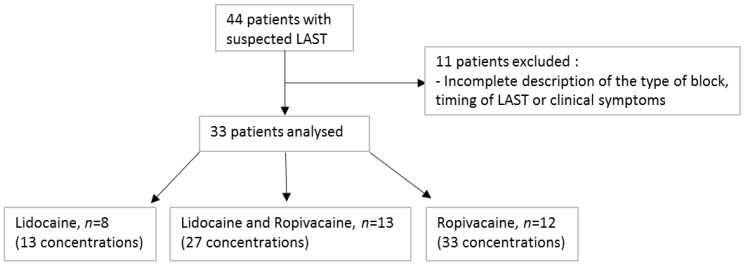
Flowchart of the study.

Thirty-three patients were included in the study, corresponding to 73 observed concentrations (Figure 1).

Patients’ ages ranged between 17 and 93 years. Approximately 82% of the patients were female, with 31% being pregnant women. Patients’ characteristics are summarized in Table 1.

The most frequent surgeries reported were obstetrics and gynecological surgical procedures (*n* = 15), followed by limb surgery (*n* = 7). One case of suspected LAST occurred during a facial block in a 47-year-old man scheduled for an adenoma pituitary resection.

The combination of ropivacaine-lidocaine (*n* = 13) was the most frequent therapeutic schedule involved in suspected LAST, followed by ropivacaine (*n* = 12) and lidocaine (*n* = 8) alone. The total injected dose of ropivacaine ranged from 0.4 to 3.0 mg/kg, and the total injected dose of lidocaine ranged from 0.7 to 8.3 mg/kg. One patient received a lidocaine dose (i.e., 8.3 mg/kg) above the maximal recommended dose for lidocaine (i.e., 7 mg/kg without epinephrine) (Supplementary Table S1).

### 3.1. Symptom Timing and Presentation

**Figure 2:**
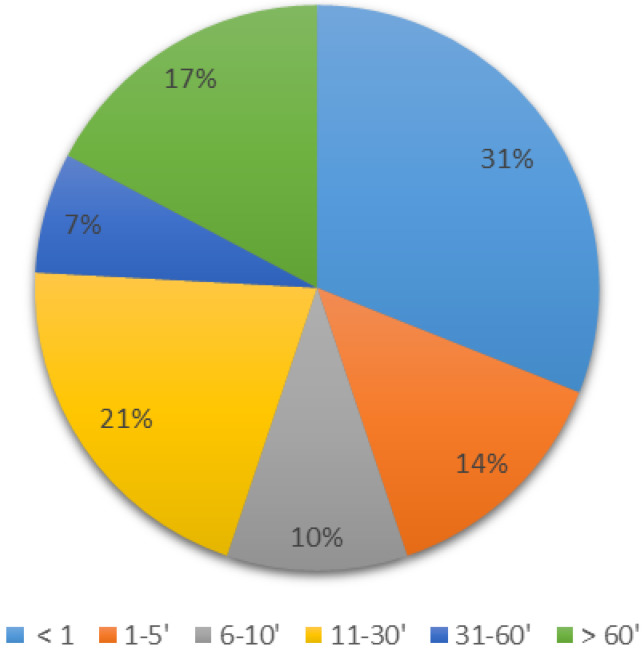
Time taken to observe clinical events after LA injections (min; *n* = 31).

The distribution of time to observe clinical events after LA injections is described in Figure 2: 55% of the reported neurologic or cardiac toxicity occurred within the first 10 min. In 31% of patients, symptoms of toxicity occurred in the first minute after injection, which is the onset delay usually assumed for direct intravascular injections (Figure 2) [6,23,24,25].

Observed signs and symptoms of CNS and cardiovascular toxicity are described in Table 2.

Prodromal symptoms of LA toxicity including light-headedness, numbness of the tongue, visual or auditory disturbances, muscular twitching, confusion, dizziness, and metallic taste were observed in 13 patients. Isolated CNS toxicity (seizure, unconsciousness) occurred in 11 patients; combined CNS and cardiovascular toxicity was reported in 12 patients; and 10 patients presented with isolated cardiovascular toxicity (cardiovascular arrest, hypotension, bradycardia) (Supplementary Table S1).

### 3.2. LA Observed Concentrations

**Figure 3:**
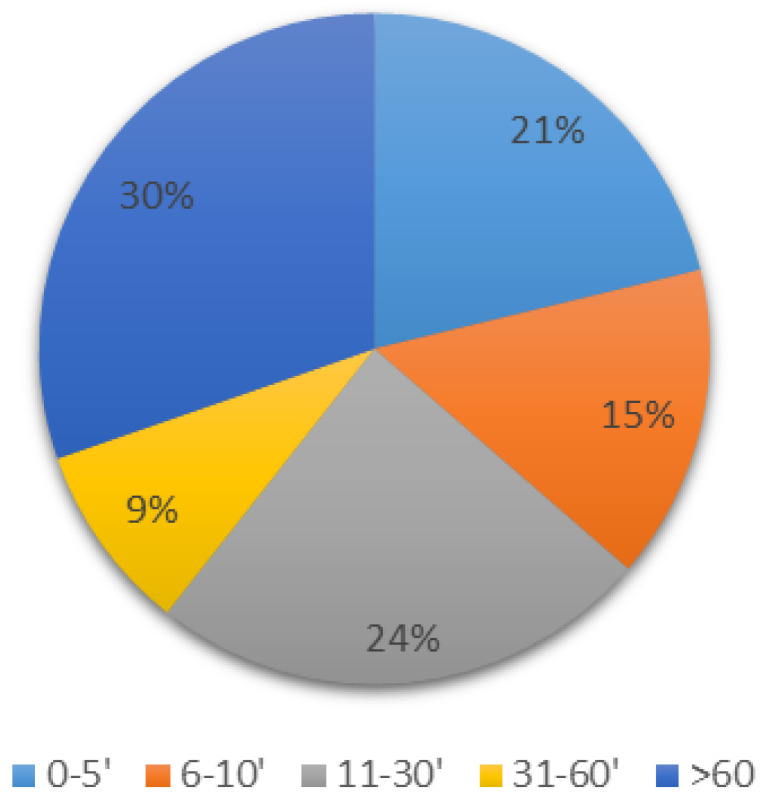
Time of the first samples relative to time of clinical symptoms (min, *n* = 33).

A single plasma sample was drawn from 11 patients, and multiple plasma samples were drawn from 22 patients. The first sample was mainly drawn in the first ten minutes after observation of clinical symptoms (*n* = 12). In 10 patients, the first samples were drawn after 60 min and were less informative (Figure 3).

Two ropivacaine concentrations above the toxic concentration threshold (4.2 and 4.8 μg/mL) were observed in the ten minutes after the observation of symptoms. In these two patients, a rapid decrease in LA concentrations was observed, which suggests an inadvertent intravascular injection. Only one lidocaine plasma concentration was observed above the common toxic threshold (16.1 μg/mL, 25 min after cardiovascular toxicity symptoms appeared) and intravascular injection was confirmed following a rapid decrease of concentrations. A large majority of reported LA plasma concentrations were less than 1.0 µg/mL for both local anesthetics, combined with a slow decrease in LA plasma concentrations (Supplementary Table S1).

### 3.3. Treatment and Patient Outcome

Intravenous lipid emulsion was used in 24 patients and was the most frequently administered treatment. In 10 patients, intravenous lipid emulsion was associated with symptomatic treatment. Cardiopulmonary resuscitation was required in two patients undergoing cardiac arrest. All the included patients recovered completely within the next few hours, and no sequela was reported. All the included patients have been informed of the medical care.

## 4. Discussion

This retrospective study illustrates the clinical spectrum of symptoms occurring after LA injection for a regional block and highlights the difficulties in detecting and diagnosing regional anesthesia complications. Typical reports of LAST consist of prodromal symptoms followed by CNS and cardiac toxicity [1,6,21], and symptoms that worsen with enhanced plasma concentrations [3,5,21]. However, 40% of reported LAST patients presented an atypical clinical spectrum [3,6]. Usually, neurotoxicity or cardiotoxicity is often a unique manifestation of LAST and/or late symptoms occur [4,6]. In our study, 30% of patients presenting only cardiovascular symptoms and prodromal symptoms were not systematically observed. Regarding the onset of symptoms, only 31% occurred in the first minute after injection, in accordance with a previous report by Vasques et al. (26% of reported LAST occurred in the first minute post-LA administration) [23].

Due to the close relationship between LAST and plasma LA concentrations, LA blood concentrations could be a relevant tool in confirming the diagnosis of LAST. However, plasma samples should be performed quickly after clinical events in order to obtain maximal local anesthetic concentration. In our study, 63% of first blood samples were performed ten minutes or more after signs and symptoms of toxicity, which could explain the small number of high observed concentrations. Indeed, we observed only three plasma LA concentrations above the usual CNS toxicity threshold. These results show the necessity of enhanced anesthetists’ awareness about the usefulness of early LA concentration determination in order to confirm LAST diagnosis. An estimation of LA peak concentration from later concentration(s) and published LA pharmacokinetics parameters has been previously proposed [26]. However, due to the flip-flop kinetic of LA, PK parameters cannot be easily estimated directly from the time curve. This strategy could probably be improved using population pharmacokinetic models and Bayesian estimation, with an accurate estimation of individual PK parameters and maximal LA plasma concentrations.

Most of the previously reported LASTs were identified as stemming from inadvertent intravascular injection and symptoms quickly occurred after block achievement [6,9,21]. During regional anesthesia, the passage of LA through blood circulation represents the route of LA elimination. In the case of partial accidental intravascular injection, a large part of the injected dose is distributed during the early phase of circulation, which produces a rapid decrease in plasma LA concentrations. Commonly, the diagnosis of accidental intravascular injection is considered if symptoms occurred within 5 min after nerve block. In Patients 8 and 16, neurological or cardiovascular toxicities occurred immediately at the end of LA injection, which corroborates with the direct intravascular injection of LA. The delay between LA injection and sampling time (40- and 45-min post-injection, respectively) probably explains the observation of plasmatic lidocaine and ropivacaine concentrations below the toxic threshold (2.2 and 1.4 µg/mL, respectively). As recommended, plasma sampling should be performed quickly after clinical events in order to observe maximal concentration and incriminate LA [22,24]. Nevertheless, this recommendation is difficult to adhere to in clinical practice and emergency medical care remains the priority in order to avoid serious and potentially permanent morbidity. In Patient 16, the initial plasma sample was collected 45 min after the onset of clinical events. However, a higher apparent elimination of ropivacaine than commonly observed in axillary plexus block confirmed the occurrence of accidental intravascular injection [18]. Additionally, in Patient 8, only two plasma samples were performed at 40 and 150 min, and LA concentrations below the toxic threshold were determined. Diagnosis of accidental intravascular injection was then confirmed for patients 8 and 16 despite nontoxic observed concentrations, as the pharmacokinetic profiles were similar to those with intravenous injections. A rapid decrease in LA concentrations was also observed in patient 19 with a ropivacaine concentration at 10 min close to the toxic threshold, which supports an accidental intravascular injection.

Excessive absorption is usually considered when delayed symptoms and toxic plasma LA concentrations are observed after injection of a significant LA dose. In order to reduce the risk of dangerously high plasma levels and the incidence of LAST, following maximum recommended dosage guidelines is necessary. Maximal doses are currently recommended according to the injection site (3 and 5 mg/kg in upper limb blocks and 4 and 7 mg/kg in lower limb blocks for ropivacaine and lidocaine, respectively) [1]. Indeed, the rate of LA absorption is related to the injection site and decreases in the following order: interpleural, intercostal, paracervical, caudal, epidural, brachial plexus, sciatic/femoral, and spinal. An excessive absorption was retained for patient 1 due to the delay in CNS toxicity (i.e., 10 min) and to the ropivacaine concentration being close to the toxic threshold observed 20 min after the onset of symptoms (i.e., 2.20 µg/mL). The patient underwent a paracervical block achieved by a mixture of low dose of ropivacaine and lidocaine (i.e., 2.5 mg/kg and 1.7 mg/kg for ropivacaine and lidocaine, respectively). The combination of ropivacaine-lidocaine was involved in approximatively 40% of suspected LAST described in this study. These results enhanced the significance to consider the additive properties of toxicity when combining two local anesthetics [27]. In patient 27, a slow decrease in LA concentrations was observed with a ropivacaine concentration close to the toxic threshold at 180 min post-injection, which also supports excessive absorption of LA in this case.

In this study, risk factors of LAST identified in some patients include pregnancy (30%), overweight (21%), and age > 60 years (18%). Several factors could explain the vulnerability of parturient women to LAST and the occurrence of LAST with or without toxic plasma concentrations in this population. An increase in sensitivity of nerve axons to neural blockage was already observed in parturient women, which enhances their risk of cardiotoxicity. Moreover, parturient women present an increased free fraction of plasma LA, which could explain the occurrence of LAST without a high level of total LA [28]. Finally, in the late stages of pregnancy, the increased cardiac output and rate of the initial uptake of LA could for allow excessive LA plasma concentrations early after the injection. In our study, we included 10 parturient women at different periods of pregnancy. Symptoms were observed immediately after injection to 90 min post-injection and no toxic LA concentrations were observed. Advanced age also promotes the occurrence of LAST because of the reduced blood flow observed during heart, liver or renal failures, which leads to a decrease in local anesthetic metabolism and elimination [29]. The subsequent increased risk for LAST in these populations requires further investigation regarding the maximum recommended dose and the toxic threshold of LA.

Some remarks need to be made regarding the observational retrospective design of this study. First, a heterogeneous cohort of patients was included in the study and important clinical data were not available, especially regarding clinical outcomes: ICU admission and need for mechanical ventilation, in-hospital length of stay, post-incident psychological morbidity or post-discharge follow-up. Moreover, co-medications were not reported; some drugs have been described to lower the convulsive threshold of local anesthetics, such as opioids [30]. Therefore, future prospective clinical studies are needed in order to improve LAST diagnosis and medical care.

To conclude, an important biological and clinical dataset of patients with suspected LAST was described in the current study. The diagnosis of LAST remains complex, even if the major mechanisms of toxicity are well described, and determination of LA concentrations could help in achieving confirmation. This study highlights that the time to perform plasma samples needs to be rigorously determined in order to contribute to LAST diagnosis. The strategy of drawing several plasma samples post-injection could improve the acknowledgement of LAST diagnosis. Finally, this protocol increases anesthetists’ awareness about the usefulness of LA plasma concentration. Further understanding of sensitivity factors remains necessary in order to prevent the occurrence of LAST.

## Figures and Tables

**Table 1:** Patients’ characteristics.

Characteristics	n (%)
Demographic	
Male/female	6/27
Body weight (kg) *	65 (45–116)
Age (years)	47 (17–93)
Risk factors	
Age > 60 years	6 (18%)
Pregnant	10 (31%)
Overweight	7 (21%)
Regional blocks	
Upper extremity	11
Torso	12
Lower extremity	9
Subcutaneous	1
Surgery	
Gynecological/obstetrics	15
Limb surgery	7
Heart	4
Others	5
NA	2
LA concentrations (*n* = 74)	
Ropivacaine	33
Lidocaine	13
Ropivacaine and lidocaine	27
Single sample	11
Multiple samples	22

* *n* = 27; NA: not available; LA: local anesthetic.

**Table 2:** Observed signs and symptoms of CNS and cardiovascular toxicity (*n* = 33 patients).

Observed Signs of Toxicity	*n*
Prodromes	13
Loss of consciousness	4
Seizures	7
Tachycardia/hypertension	5
Bradycardia/hypotension/shock	7
Cardiac arrest	3
Ventricular tachycardia/ventricular fibrillation	3
Ventricular ectopics	1
Bundle branch block	1

## Data Availability

The data that support the findings of this study are available on request from the corresponding author upon reasonable request. The data are not publicly available due to privacy regulations.

## Supplementary Materials

The following supporting information can be downloaded at: https://www.mdpi.com/article/10.3390/pharmaceutics14040708/s1, Table S1: Individual patient characteristics and suspected LAST description.
