# Supplementary material for: Local Anesthetic Plasma Concentrations as a Valuable Tool to Confirm the Diagnosis of Local Anesthetic Systemic Toxicity? A Report of 10 Years of Experience
Source: Pharmaceutics. 2022 Mar 26;14(4):708. doi: 10.3390/pharmaceutics14040708 (PMC9025106; doi:10.3390/pharmaceutics14040708)
Supplement: Supplementary file 1 [file pharmaceutics-14-00708-s001.zip › pharmaceutics-1568346-supplementary.pdf]

pharmaceutics

MDPI

Supplementary Materials: Local Anesthetic Plasma Concentrations as a Valuable Tool to Confirm the Diagnosis of Local Anesthetic Systemic Toxicity? A Report of 10 Years of Experience

Camille Riff, Axel Le Caloch, Julien Dupouey, Laurent Allanioux, Marc Leone, Olivier Blin, Aurélie Bourgoin and Romain Guilhaumou

Table S1. (Part 1). Individual patient characteristics and suspected LAST description.

							Plasma concentration			Plasma concentration		Side effects					
Patient	Sex	Age (years)	Weight (kg)	Surgery/Procedure	Block	Ropivacaine dosage (mg.kg-1)	Delay to time of symptoms (min)	Total (mg.L-1)	Lidocaine dosage (mg.kg-1)	Delay to time of symptoms (min)	Total (mg.L-1)	Neurologic	Cardiovascular	Time after injection (min)	Risk factor	Treatment	Evolution
1	F	39	59	fibroma resection	Paracervical	2.5	20	2.2	1.7	20	2.1	Limb tonic-clonic seizure, loss of consciousness	Tachycardia/hypertension	10	-	IL	recovery within 10 minutes
2	F	33	51	hand surgery	Axillary	2.6	25	1.3	6.9	25	4.4	Nausea, confusion	Hypotension (following CNS toxicity)	10	-	IL, midazolam	resolved with treatment
3	M	22	75	knee ligamentoplasty	Femoral	1.3	110	0.66	-	-	-	-	Atrial-ventricular block	70	-	IL, ephedrine, atropine	resolve with therapies
4	F	92	50	transtibial amputation	Femoral	3.0	10	0.66	-	-	-	-	Ventricular fibrillation, cardiopulmonary arrest	15	Age > 60 years	IL, adrenaline, external defibrillation	recovery after rescuscitation manœuvres
5*	F	30	64	foetoscopy	Epidural	-	-	-	1.4	30	1.3	-	Cardiovascular collapse	immediately	Pregnant (2nd trimester)	IL, vascular filling, ephedrine, adrenaline,	recovery without sequelae
6	M	81	**	bimaleolar ankle fracture	Femoral and sciatic	225***	5,90,210	4.2,1.8,1.0	-	-	-	Seizure , loss of consciousness	Tachycardia, hypotension	15	Age > 60 years, know cardiac	IL, midazolam	**
7	M	17	45	**	Femoral	0.9	90,18	0.19,0.09	-	-	-	-	Ventricular tachycardia	300	Small weight, sudden death history	**	self-limited
8	F	94	60	**	Ilio-facial	-	-	-	5.0	40,150	2.2,1.3	Lost of consciousness	-	immediately	Age > 60 years	**	recovery within 10 minutes
9*	F	22	53	caesarean	TAP	2.8	0,105,285	4.8,2.4,1.2	-	-	-	Confusion loghorrea, cephalalgia	-	20	Pregant (3rd trimester)	IL	resolve 30 minutes after IL injection
10	F	53	73	laparoscopic hysterectomy	TAP	2.0	35,95,215	1.1,0.6,0.52	-	-	-	-	Cardiovascular collapse	25	Overweight	Vascular filling, phenylephrine	resolve with vascular filling
11*	F	41	91	delivery	Epidural	1	15,75,165	0.66,0.56,0.37	-	-	-	Confusion	-	30	Overweight	IL	recovery a few minutes after IL injection
12	M	47	60	adenoma pituitary	Intranasal	-	-	-	8.3	25,60,180	16.1,5.35,3,73	-	Bradycardia and hypotension, followed by hypertension and ventricular ectopics	5	-	Vascular filling, phenylephrine	**
13*	F	32	84	induced abortion	Paracervical	0.5	20,50,200	0.51,0.34,0.19	2.4	20,50,200	1.01,0.99,0.49	Tonic clonic seizure, bilateral mydriasis	-	10	Pregant (1st trimester), overweight	IL	general anesthesia was induced secondarly to agitation, no sequelae
14	F	50	55	fibroma resection	Paracervical	0.7	0,60,260	0.29,0.12,0.1	3.6	0,60,200	0.58,0.44,0.26	Metallic taste	Bradycardia, hypotension	100	-	IL	quick recovery
15	F	31	52	endocervical curettage	Paracervical	0.8	10,60,210	0.24,0.1,0.09	3.8	10,60,210	1.09,0.9,0.56	-	Cardiac arrest	20	-	Atropine, cardiac massage, adrenaline	resolved with adrenaline
16	M	31	68	arthrodesis of scaphoid	axillary	2.2	45,180,300	1.4,1.0,0.44	-	-	-	Metallic taste, tinnitus, hypotension, Bradycardia	Bradycardia, hypotension	immediately	-	IL, atropine	recovery several minutes after IL injection

Lid: lidocaine; Rop: ropivacaine; TAP: transverse abdominis block; IL: Intralipid
* pregnant woman; ** not documented; ***dose in mg

Table S1. (Part 2). Individual patient characteristics and suspected LAST description.

							Plasma concentration			Plasma concentration		Side effects					
Patient	Sex	Age (years)	Weight (kg)	Surgery/Procedure	Block	Ropivacaine dosage (mg.kg-1)	Delay to time of symptoms (min)	Total (mg.L-1)	Lidocaine dosage (mg.kg-1)	Delay to time of symptoms (min)	Total (mg.L-1)	Neurologic	Cardiovascular	Time after injection (min)	Risk factor	Treatment	Evolution
17	M	20	89	arteriovenous fistula	Axillary brachial plexus	0.85	5,360,	0.05,0.15	1.1	5,360	0.05,0.08	Agitation, Metallic taste	Cardiovascular collapse	immediatly	Overweight	IL	quick recovery
18	F	36	98	conization	Paracervical	0.4	10,60,120	0.32,028.0,12	4.0	10,60,120	1.4,1.4,0.54	Agitation	-	5	Miscarriage	IL	quick recovery
19	F	47	**	conization	Paracervical	-	10,60,180	2.0,0.24,0.13	400***	10,60,180	0.56,1.5,0.42	Perioral paresthesias tinnitus, metallic taste	Tachycardial	1	-	IL , O2	quick recovery
20	F	93	**	endarterectomy	Paracervical	60***	10	1.0	40***	10	0,5	Brief seizure	-	60	Overweight	IL	quick recovery
21	F	44	65	chest tube	Paracervical	0.6	5	1.1	2.6	5	3,1	Limb tonic-clonic	-	immediatly	-	IL, midazolam	recovery within 10 min
22*	F	35	82	stillbirth	Epidural anesthesia	-	-	-	1.2	15,75,195	0.85,0.42,0.23	-	Bradycardia	immediatly	Miscarriage	atropine	quick recovery
23*	F	18	50	delivery	Epidural anesthesia	**	150	0.77	2.0	150	0.79	Agitation, myoclonus	Tachycardia, hypertension	1	Pregnant (3rd trimester)	**	**
24*	F	36	58	delivery	Epidural anesthesia	-	-	-	1.4	60,120,240	0.55,0.25,0.18	Tonic-clonic seizure	Tachycardia, hypotension	immediatly	Pregnant (3rd trimester)	IL, adrenaline	recovery after IL
25*	F	31	89	delivery	Epidural anesthesia	-	-	-	0.7	120	1.1	Tonic-clonic seizure	-	immediatly	Pregnant (3rd trimester), overweight	IL, clonazepam	**
26*	F	40	85	delivery	Epidural anesthesia	-	120,180,240	0.28,0.24,0.34	3.5	120,180,240	0.26,0.2,0.77	Nausea, confusion, dyzziness, myoclonus	-	90	Pregnant (3nd trimester)	O2	recovery within 10 min
27	F	68	**	appendectomy	Intercostoiliac	60***	5,120,180	2.2,2.9,2.9	-	-	-	Confusion, hemiplegia	Tachycardia	180	Age > 60 years	IL	**
28*	F	39	116	caesarean	Spinal	1.6	60,120,24	0.13,0.08,0.06	-	-	-	-	Bradycardia, hypotension	**	Pregnant (3rd trimester), overweight	ephedrine , O2	**
29	M	23	80	knee ligamentoplasty	Femoral	0.9	75,135,255	0.32,0.23,0.36	-	-	-	Myoclonus	-	immediatly	-	IL	**
30	F	84	**	femoral neck fracture	Femoral	150***	300,360,480	1,0,1.5,1,0	-	-	-	Tonic-clonic seizure	Ventricular fibrillation	**	Age > 60 years	IL	**
31	F	30	64	bartholin gland cyst	Pudental	0.94	0,60,180	0.52,0.22,0.23	-	-	-	Seizure	-	45	-	IL, clonazepam, propofol	recurrence of symptoms
32	F	91	50	vascular surgery	Ilio inguinal	1.1	195	0.66	**	195	1.2	Loss of consciousness	Hypotension	135	Age > 60 years	IL	quick recovery
33	F	59	**	tibial melanoma	Subcutaneous	-	-	-	50***	15	2.7	-	Dysarthria	5	-	IL	**

Lid: lidocaine; Rop: ropivacaine; TAP: transverse abdominis block; IL: Intralipid
* pregnant woman; ** not documented; ***dose in mg
